# Supplementary material for: Sex-specific tuning of modular muscle activation patterns for locomotion in young and older adults
Source: PLoS One. 2022 Jun 3;17(6):e0269417. doi: 10.1371/journal.pone.0269417 (PMC9165881; doi:10.1371/journal.pone.0269417)
Supplement: Attachment — Submitted filename: Response to Reviewers.docx [file pone.0269417.s007.docx]

Reviewer #1

**The present manuscript uses the non-negative matrix factorization technique to assess potential differences in synergistic muscle activation patterns in walking and running between men and women as well as between age groups (walking only). The analysis of data obtained in a sample of 215 subjects revealed minor differences in the contribution of hip and knee extensors in various synergies (as reflected by motor modules) and in the timing of synergistic muscle activity (as reflected by primitives).**

**By studying the effect of gender on motor control, this paper addresses an important and still understudied subject. Personally, I have never worked on muscle synergies and had to read up on the non-negative matrix factorization technique. Given my lack of experience, I am not qualified to judge the methodological procedure but understand that it represents the preferred synergy extraction method (Rabbi et al., 2020, Sci Rep). I was much impressed by the apparent efforts the authors have put into data acquisition (a sample size of 215 is way beyond the limits typically seen in basic biomedical research in humans in vivo), analysis and presentation. In this regard, I would like to sincerely thank the authors for also providing their raw data and R-code, which I found to be both instructive and very elegant.**

We thank the reviewer for their flattering words and are especially enthusiast about the words on the open access nature of our data and code. The responses to specific comments are detailed below.

**In terms of critique, I can only mention that I found the shear amount of information presented difficult to absorb. I also expect other readers not familiar with motor control research and, specifically, techniques for the extraction of muscle synergies to have a hard time going through roughly 20 manuscript pages and a total of no less than 14(!) figures, trying to grasp the central and most important findings.**

We endeavored to simplify the presentation as detailed in the next points and thank the reviewer for this important comment.

**With regard to figures, please also note that in some cases the legends are numbered incorrectly and do not match the respective figures.**

We thank the reviewer for this: the order has been reviewed and follows the logic detailed below.

**With simplicity in mind, I would have preferred if synergy results had only been reported for those muscles that are primarily responsible for the execution of a given phase of the gait cycle. This is because I am wondering whether differences in the synergy of muscle activity are physiologically relevant if the gross activity of the respective muscles is very low to start with. In figures 4 and 5, vertical lines or boxes might be introduced to highlight the prime movers of each phase. Similarly, in figure 5b the co-activations of muscles with little activity (the ones shown in semi-transparent colors) could be entirely omitted.**

The question whether some muscles are prime movers or not is profoundly relevant in muscle synergy analysis. Ultimately, it boils down to quantifying the relative contribution (i.e., the motor modules) of each muscle compared to the others and to the maximum. While we would prefer not to address this very relevant yet insidious topic in the manuscript, we do agree with the reviewer that some sort of highlight in the figures could be of help to the reader of any experience level. Now the figure features some boxes that highlight the most physiologically relevant muscles for each synergy. With simplification in mind and agreeing with the reviewer that the amount of information is high, we also moved Figure 4 to the supplemental information (now Figure S2).

**Finally, I would have loved to see a bulleted list (maybe at the end of the Discussion) summarizing the most important sex- and age-specific differences in the degree and timing of muscle synergies.**

This very nice idea has been now implemented in the discussion (line 288 and following):

“*With those limitations in mind, the main results can be summarized as follows:*

- *Neither sex or age had large effects on stance, swing or cadence during walking or running*
- *Relative muscle contributions (i.e., motor modules and coactivations) were largely unaffected by sex and age, with minor and sparse differences*
- *Muscle activation motifs (i.e., motor primitives) were wider in younger males in the propulsion synergy (walking) and in the weight acceptance synergy (running); in older adults, this difference disappeared*
- *The complexity of motor primitives for walking was affected by sex in older (i.e., lower in females) but not in younger adults.*”.

**Other than that, I would like to congratulate the authors for their beautiful work, which will certainly be of interest to the readership of PLOS ONE.**

We thank one last time the reviewer for their congratulations and relevant comments, that surely improved the quality of the manuscript.

Reviewer #2

**General comments:**

**The paper is well written and it is easy to follow the lines of argumentation. The authors address an important issue, by pointing out the lack of studies involving or focusing on female participants in clinical research. By applying the muscle synergy concept, the authors aimed at drawing conclusions about possible sex-dependent differences in muscle coordination patterns out of their analysis. An additional aim was to analyze whether these differences are maintained in older age. Thereby, the authors do not solely focus on the muscle synergy results but also take the analysis of spatio-temporal parameters into account.**

**To do so, the authors combined data from three different data collections (G1, G2, G3). For me, it is not clear whether these data have been published before?**

We thank the reviewer for their careful critique. Please find below our replies to the specific comments. Concerning the data sets, they have only partially been published before. This has been now specified in the text as follows (line 356 and following): “*Part of the presented data sets have been previously published: G1 partially in [31,41,47,59]; G2 (unpublished); G3 partially in [31,41,47,59,60].*”.

**As far as I understood the manuscript, G1 and G2 were used to analyze the differences between females and males during walking, ones in younger age (G1) and ones in older age (G2). G3 was used to analyze the differences in running in younger age. It is mentioned that both fixed and self-selected speeds were chosen and that this information is included in the supplemental material. However, I think the information about whether the speeds mentioned in Table 1 were self-selected or prescribed should also be included in the manuscript.**

The reviewer is correct in their summary. Within each group, we had a mix and match of preferred or fixed speeds, due to data collection in different experimental setups. For this reason, we decided to not overcrowd Table 1 with information about speed type and refer the reader to the external repository where all the metadata are safely stored. This is specified as (line 324 and following): “*The participants were asked to walk or run at either their preferred speed […] or at a fixed speed chosen to be close to the average preferred speed, depending on the group [57,59] (details are given in the supplementary information at Zenodo [56])*”. At the repository, a file in version 1.2.0 called “metadata.dat” (link for the download: <https://zenodo.org/record/5171823/files/metadata.dat?download=1>) contains a column called “Speed_type” wich makes the distinction between fixed (decided by the researchers) or preferred (selected by the participant) speed.

**The motor synergies were compared using both a linear (duration of activity and point in time of main activation) and a nonlinear metric (local and global complexity of the signals). The authors choose to calculate Bayesian statistics. Unfortunately, I am not familiar with Bayesian statistics and I am thus not able to comment on this part of the manuscript.**

**I was not sure about some parts in the presentation of the results of the gait cycle parameters. These points are pointed out below. Four synergies have been determined, which account for 80-85% of the explained variance. Is there a specific reason that the authors did not choose the common threshold of 90%, which might have led to five synergies?**

As the reviewer correctly pointed out, in our usual synergy extraction approach we do not impose a threshold on the reconstruction quality. We are of the opinion that constraining the global and/or the muscle-specific reconstruction quality to fixed values would be less physiologically meaningful than using the linearity method employed here, which originates from the work of other authors [1]. Assuming that biological systems work with “good-enough” strategies rather than searching for optimal ones [2], setting a threshold would constrain this fundamental freedom. Instead, the method proposed by Cheung and colleagues searches for a number of synergies after which one more synergy would not add much new information to the model, in other words declaring it as “good enough”, thus aligning better to our physiological perspective on the matter.

**Generally, the authors could consider highlighting of large effects in the figures.**

While we appreciate the sense of this comment, we would like to stick to the nature of the statistical framework employed, which by nature would attempt to avoid guiding the conclusions of the readers by using visual or numerical hints. We believe that putting together the Bayesian approach and a more classical linear mixed modelling gives enough freedom for interpreting of the results.

**There are some minor points concerning the discussion section, which are pointed out below. I am though missing a paragraph referring to the limitations of the study, e.g. the three different procedures and the various walking/running speeds.**

This was certainly missing and we are happy to having added some comments to the discussion (line 279 and following): “*The presented results must be interpreted with an eye to the limitations of the design. Participants were not tested in the same experimental environment and three distinct laboratories and treadmills – but the same EMG system – were used to gather the data. Moreover, due to different experimental setups, some participants walked or ran at their preferred speed, while others were asked to complete the locomotor task at speeds that were predetermined by the researchers. While the difference between preferred and predetermined speeds were small, we cannot exclude an effect on our results. Last but not least, the groups were formed to be as homogeneous as possible. Yet, small to large effects of sex were observed in both age and body mass index, to which the presented results were not normalized.*”.

**Specific comments:**

**Results-section: It might be easier to follow if the results for the gait cycle parameters would be better separated in results concerning walking and results concerning running.**

We gladly rearranged the results section to accommodate this comment and agree with the reviewer that now readability improved. The paragraph now read as follows (line 93 and following): “*During walking, the duration of* *the stance phase (Figure 1 and Figure 2A) was moderately affected by both sex (β = -35.57, CrI = [-58.65, -12.33]) and age (β = 15.95, CrI = [-1.04, 32.79]), with an interaction between the two. The post-hoc analysis revealed a stronger effect of sex on the stance duration in older adults, while in both age groups females spent less time than males in the stance phase (Figure S1). It might look like the different walking speeds played an important role; however, this effect was associated with a large estimation uncertainty, as visible from the large width of the credible interval (β = 160.86, CrI = [52.90, 269.44]). Specifically, the walking speed in young adults ranged between 1.1 and 1.5 m/s, while in older adults it spanned between 1.0 and 1.4 m/s (see also Table 1). The swing duration (Figure 1 and Figure 2B) was moderately affected by both sex (β = -16.8, CrI = [-34.01, 0.34]) and age (β = -15.66, CrI = [-28.22, -3-31]), with negligible interaction between the two. Regardless of age, females showed shorter swing duration than males, while older adults had shorter swing times than younger participants. For cadence, or number of steps per minute (Figure 1 and Figure 2C), there was only a small (if any) effect of sex (β = 0.44, CrI = [-2.98, 3.92]) and age (β = -1.98, CrI = [-4.52, 0.61]). The considerations about the effect of walking speed are also valid for the swing times (β = 62.94, CrI = [-14.78, 142.34]) and cadence (β = 13.21, CrI = [-3.30, 29.41]). In running, sex had a negligible effect on the stance times (β = 1.48, CrI = [-15.22, 18.76]) and the different locomotion speed was also of little importance (β = -2.95, CrI = [-30.31, 25.03]). Running speeds ranged between 2.0 and 3.8 m/s (see also Table 1). Swing duration was not clearly affected by sex (β = 5.41, CrI = [-13.10, 25.19]), but the locomotion speed had a moderate effect on this parameter (β = 31.88, CrI = [1.25, 64.25]). Running cadence showed a moderate effect of sex (β = 4.03, CrI = [-0.32, 8.47]) and speed (β = 4.65, CrI = [-2.59, 12.06]).*”.

**line 97: Figure 1 or S1?**

In our file, we indeed read “Figure S1”, which is the correct cross-reference.

**lines 104ff: “The swing duration […] was moderately affected by both sex […] and age …” does this refer to running? If yes, this is somewhat contradictory to line 107/108: “In running, swing duration was not clearly affected by sex”**

As mentioned above, the paragraph was indeed confusing and has now been rearranged.

**line 132: The modules can also be seen in Figure 4? Figure 4 and Figure 5 are in general somewhat redundant – can’t they be combined?**

We agree with this comment that mirrors a request from Reviewer #1. For this reason, we moved Figure 4 to the supplemental information and highlighted the prime contributors to each synergy in Figure 5 (now Figure 4) to improve readability.

**lines 133/134: I am not sure about the statement(s) that one or the other muscle contributes more or less – mustn’t we consider both the module(s) and the primitive(s) in order to make this statement?**

Since motor modules are the time-invariant coefficients (also called weights or weightings), they represent the contribution of each muscle, relative to the others, to a specific synergy. Certainly, motor primitives play the role of describing how this relative contribution is evolving over time, but the meaning of our sentence was to address the synergy-specific contribution. We rephrased this part as follows (line 129 and following): “*By looking at the motor modules (Figure 4, Figure S2 and Figure S3), it is possible to identify the contribution of each muscle, relative to the others, to a specific synergy*”.

**line 148: Figure 5B?**

As mentioned above and below, figures have been rearranged and incorrect cross-references checked for plausibility.

**lines 216ff: Considering the muscle-synergy-studies about walking and running, are there any having female participants? If yes: could the results/tendencies found here be linked to previous results concerning the muscle synergies in males in females (even though no direct comparisons have been made)? If no: this could be emphasized as novelty (besides the analysis of sex-specific differences).**

Not that we are aware of. Moreover, we would like to refrain from making novelty claims in the main text, as we believe that we sufficiently addressed the importance of including sex as a biological variable in future studies.

**lines 227ff: Could you please point out more specifically what you would like to say with this sentence?**

We thank the reviewer for pointing out a potential obstacle to fluent reading. Here, we claim that the differences in motor modules due to sex might be useful information for designing training interventions that are specific for females or males. Yet, these differences are “sparse”, thus caution is needed in their interpretation. We reformulated the sentence as follows (line 226 and following): “*The identified sex-specific contributions of the hip, knee and ankle joint muscles to the relevant synergies were sparse. However, if further investigated, they could potentially be useful for the design of locomotion-based experimental protocols or training interventions that account for the biological sex of the participants.*”.

**line 247: Which past results do you refer to?**

A poor choice of words, as we meant the current study presented here. We rephrased as (line 245 and following): “*An explanation of this phenomenon cannot be directly supported by our results, but it is possible to speculate on its potential neurophysiological importance based on present and past outcomes.*”.

**lines 258ff: I am sorry, but to me, the connection is not clear.**

**lines 261ff: Do you have a possible explanation for this contradiction?**

Here we state that, despite shorter activation, literature tells us that females are not more economical neither in absolute nor in relative (i.e., normalized by the body mass) terms and clearly an explanation cannot be supported by the present data. We rephrased as (line 255 and following): “*Our results show that muscles were being activated for a shorter amount of time in females than in males during the stance phase of locomotion, when muscle activities are at their highest (and so are energy requirements). Yet, it is known from previous studies that, in elite athletes running at similar submaximal speeds, males are more economical than females in terms of absolute oxygen uptake and no sex specificity exists when the oxygen uptake is normalized by the body mass [51]. Thus, the shorter activation does not translate in lower energy consumption, likely due to the myriad of other variables that come into play.*”.

**line 275: “are not be necessarily”: I think there is a typo.**

Indeed, it has been corrected (line 276).

**lines 307ff: Maybe you could mention which treadmill belongs to which dataset.**

This observation made us realize we did not specify in the manuscript which treadmill was used in which group. It is now clarified in the text as follows (line 333 and following): “*Data collection for the participants of group G1 was conducted on treadmills a) (33 participants, 16 females), b) (18 participants, 7 females) and c) (19 participants, 12 females); for group G2 on treadmill c); for group G3 on treadmills a) (105 participants, 53 females) and b) (15 participants, 7 females).*”.

**lines 318ff: Have you considered including adductor muscles? These might be interesting for sex-related differences (even though hard to derive).**

Yes we did and, in fact, the *adductor longus* has been part of our experimental setup in previous studies [3–5]. However, we do not include this muscle any more for two main reasons: 1) as seen in our past results, adductors do not play a prime role in any of the synergies and are contributing to many phases of the gait cycle in low amount relative to other muscles; 2) especially in females and older adults, adductors are difficult to record from given the small available space between the two upper legs, especially in overweight populations; this creates a lot of movement artefacts that are extremely hard to get rid of and negatively impact the signal. Thus, we prefer to exclude this muscle group from our standard setup.

**lines 331ff: Have you evaluated the influence of the three different segmentations on a sample data set?**

Yes, we reported the validation results in the supplemental information of one of our previous publications [6] (page S5): “*Both the approaches for the determination of touchdown and lift-off have been validated using force plate data (AMTI BP600, Advanced Mechanical Technology, Inc., Watertown, MA, USA) from 15 participants walking and running overground at six different velocities without perturbations and from the data recorded in G1 overground. We then calculated the true errors between the contact times detected via force plate and those obtained from the acceleration data and used the averages to correct the calculations. Errors were of 1.9 ms for touchdown and 13.1 ms for lift-off in walking and -4.1 ms and -13.2 ms for running.*”. Moreover, this procedure has been mentioned in the present manuscript as well (line 366 and following): “*Both algorithms were validated against data recorded from 30 young adults (15 females, height 173 ± 10 cm, body mass 68 ± 12 kg, age 28 ± 5 years) during walking and running (at 1.4 m/s and 2.8 m/s, respectively) over a 900 x 600 mm force plate (1 kHz, AMTI BP600, Advanced Mechanical Technology, Inc., Watertown, MA, USA), with ten steps recorded per participants, for a total of 300 steps for walking and 300 for running.*”.

**line 348: What is the unit of the stance and swing times?**

We specified that units are milliseconds, the same displayed in the figures (line 374).

**line 356: Have you averaged the 30 cycles before applying the NMF algorithm? Earlier, you mention differences in gait cycle parameters. You normalize the stance and the swing phase separately. This can influence your results, you can lose some (timing) information - could you comment on that?**

Only after NMF we calculated the relevant metrics and then averaged the obtained values. This is detailed at lines 381 (for the full width at half maximum) and 384 (for the center of activity). Concerning the normalization, we agree that absolute timing information is lost. Yet, relative time information is gained and that is the goal of our normalization approach. In other words, we are keener to comment on the relative duration with respect to the stance and swing phase separately, rather than to the whole gait cycle. This choice allows us to compare the relative duration of gait phases that might be extremely different, such as those of young and older adults in walking or those of walking and running.

**lines 376ff: Even though these methods are properly referenced, a motivation why these methods add value, since they are not commonly used (to the best of my knowledge), might be helpful for the reader. E.g., an explanation of how certain values of these metrics might be interpreted in a muscle synergy context.**

While the full width at half maximum and center of activity are widespread metrics for the analysis of motor primitives, we agree that fractal analysis is a novel approach in the framework of muscle synergies. We thank the reviewer for this request, which we gladly satisfy. Part of the text in those paragraphs was in fact written to better explain the added value of using those metrics. We now reworded the section as follows (line 405 and following): “*The full width at half maximum is used to describe the duration (i.e., the width) of motor primitives relative to the gait cycle and it was calculated cycle-by-cycle after subtracting the cycle’s minimum as the number of points exceeding each cycle’s half maximum, and then averaged [43]. The center of activity, an indication of when the main activation is happening in time, was also calculated cycle-by-cycle as the angle of the vector in polar coordinates that points to the center of mass of the circular distribution defined between 0 (touchdown) and 360° (next touchdown) and then averaged [45]. The advantage of using the center of activity instead of the global maximum is that the former can better locate the timing of main activity when primitives are “rough” and several local maxima approaching the value of the global maximum are present in the signal [45]. To assess the local and global complexity of motor primitives, we calculated the Higuchi’s fractal dimension and Hurst exponent, respectively [69,70]. The local complexity can be seen as a measure of “roughness” (or noise content) in the signal within each gait cycle (thus the term “local”), while the global complexity is a measure of how “accurate” each cycle’s activations are, when compared to the others of the same trial (thus the term “global”).*”.

**lines 479 - 498: I think up from here your captions are somehow mixed up.**

We found in fact a repetition in the caption of Figure 5 that has been now deleted. We double-checked all the other captions and, as stated above, assigned a new order to main and supplementary figures.

**I guess “s.d.” in Figure 5(B) signifies “standard deviation”?**

Yes, and the definition has been now added to the caption for clarity.

Reviewer #3

1. **It is critical to calculate the sample size and power when designing the study. Based on a frequentist t-test, the sample size in G3 may only detect a medium effect size. I am not convinced that the study is sufficiently powered, especially for testing the interaction between sex and age.**

We would like first of all to thank the reviewer for their in-depth analysis of our statistical approach. The manuscript benefited very much from the needed review. Below we endeavored to address the reviewer’s concerns.

Regarding the power, the reviewer is right in that the detected effect size would be medium if one wanted to proceed with a frequentist power analysis *a posteriori*. However, we still find some relevant effects in our data (retrospective power analyses are known to be misleading and a focus on the effect sizes should be preferred [7]). Moreover, a prospective or *a priori* power analysis would have been in our case of difficult implementation due to two main reasons: 1) the scarcity of previous literature; 2) the non-frequentist statistical framework we chose. While the first limitation might be bypassed by making some assumptions, the second is still dependent on the lack of consensus [8,9]. At any rate, we always analyze the sensitivity to the priors of our models before finalizing our choices. In this data set, all parameters are largely insensitive to priors, something we commonly check by testing the final models against the same models implemented with uniform and with antagonistic priors (i.e., having the same shape but located on the opposite of the effect). We don’t aim at overcrowding the manuscript with the results of this very specific kind of analysis. Nonetheless, we are happy to bring an example to the reviewer. In the following figure, we show the estimated posterior distributions for the effects of speed and sex on the parameter “swing” (swing time in milliseconds) for running in younger adults. It is noticeable that the two predictions, one with flat and the other with informative priors, are extremely similar. This is mostly due to the fact that our data sets are built on a rather high number of participants, making data points not quite sparse. In conclusion, we believe that this kind of statistical analysis does make justice to the sample size and from a Bayesian perspective we cannot conclude that the study is underpowered.

Figure 1. Posterior distributions for the gait parameter swing calculated for running in younger adults. Informative (left) and flat (right) priors were used for estimating the two outcomes.

2. **The determination of prior information needs to be justified or citied references.**

Indeed, this was not sufficiently documented. We now report our sources and criteria as follows (line 456 and following): “*We used different priors depending on the investigated parameters and based on previously published data [31,47,50,59,60], without distinction of sex or age and doubling the standard deviation for normal distributions and using scale θ = 0.5, shape k = mean / θ for gamma distributions.*”.

3. **Did you test the interaction between speed and age, and speed and sex?**

We thank the reviewer for this important question. No, we did not test for those two additional interactions to avoid further complicating the testing design. This is explicitly reported in Eq. (1) and (2) and relevant text (line 443 and following): “*The constant effects analyzed were: sex, age and their interaction in G1 and G2 (Eq. 1); sex in G3 (Eq. 2). Locomotion speed was modeled as a covariate in all groups, to account for the different speeds between groups and/or participants. As random effects, we considered by-participant random slopes for the effect of the independent variables. The two models can be respectively summarized with the following equations:*

*y ~ speed+sex*age+(1|participant)(1)*

*y ~ speed+sex+(1|participant) (2)*

*where y is the analyzed dependent variable.*”. While it might be of interest for future developments, we do not believe that further interaction tests could add such value to the current manuscript to deserve further analysis.

4. **Age should be compared between sexes for each group to ensure the balance of age. BMI may need to compared too.**

We now report the differences in age in percentage and the relevant effect size (line 320 and following): “*Details about the participants and locomotion speeds are given in Table 1: females were younger (10.0% in G1, Cohen’s d = -0.72; 2.6% in G2, d = -0.40; 6.3% in G3, d = -0.37) and had lower body mass index (9.6% in G1, d = -0.86; 5.9% in G2, d = -0.53; 6.5% in G3, d = -0.65) than males.*”. We were happy to add something about this in a new section about the study’s limitations: “*The presented results must be interpreted with an eye to the limitations of the design. Participants were not tested in the same experimental environment and three distinct laboratories and treadmills – but the same EMG system – were used to gather the data. Moreover, due to different experimental setups, some participants walked or ran at their preferred speed, while others were asked to complete the locomotor task at speeds that were predetermined by the researchers. While the difference between preferred and predetermined speeds were small, we cannot exclude an effect on our results. Last but not least, the groups were formed to be as homogeneous as possible. Yet, small to large effects of sex were observed in both age and body mass index, to which the presented results were not normalized.*”.

5. **There are too many figures.**

We thank all reviewers for their efforts in indicating the excessive number of figures. We now moved one of the two synergy figures to the supplementary information and added to the former Figure 5 (now Figure 4) some boxes to identify primary contributors as suggested by Reviewer #1.

6. **Figure 1, 6 and 8 can be simplified. Just show either boxplot or dotplot, but definite not three types altogether for each outcome. The current boxplot does not seem correct, as outliers outside 1.5* interquartile range were not shown.**

With due respect to the reviewer’s opinion, we would prefer to keep the figures as we initially submitted, since the three kinds of display are complementary and each adds information that could be useful to the reader. As pointed out by Krzywinski and Altman [10], boxplots are certainly superior to other representations such as barplots with error bars. Yet, boxplots alone might still obscure the underlying distribution and violin plots might be a viable, yet somewhat redundant in that they present twice the same information, alternative [11]. Showing the individual trials as dots alone is not the best solution either, since it is hard for the reader to guess the underlying distribution of the data. For these reasons, we believe that following the suggestion of Allen and colleagues [11] and reporting all three data formats is the best solution to be completely transparent with the reader about the results.

As for the whiskers of our boxplots, they were plotted in Tukey’s style, so that the whiskers extend between [-1.5 * IQR; +1.5 * IQR]. Where outliers were present, they were indeed plotted outside this range.

7. **I had a hard time to understand the figures for effect size. The distribution figure is not necessary as almost all of the posterior distributions seem to be normal. Consider present a forest plot with effect sizes and 95% credible intervals.**

While most of the posterior distributions look like quasi-normal, we would prefer to keep the plots as they are since they clearly show the difference between a confidence interval and a Bayesian credible interval, where not only a family of values but also their probability distribution can be calculated [12]. We support this way of presenting results to give the reader the most complete set of tools to draw their own conclusions, without omitting too many details about the underlying data.

8. **Does “Sex:Age” refer to the interaction between sex and age? What does a negative/positive effect size of interaction mean? It seems that none of the interactions are significant. However, it is not conclusive whether the interactions are really not significant or the study is lack of power to detect the significance.**

The reviewer is correct in their guess. The sign is that of F – M (this is stated in the figure legends) and Young – Old. Given the second part of this comment, we would prefer to not crowd the figure legends with this additional information. Concerning the potential lack of power, we hopefully address the question by answering the first comment.

**References**

1.	Cheung VC-K, D’Avella A, Tresch MC, Bizzi E. Central and sensory contributions to the activation and organization of muscle synergies during natural motor behaviors. J Neurosci. 2005;25: 6419–34. doi:10.1523/JNEUROSCI.4904-04.2005

2.	Loeb GE. Optimal isn’t good enough. Biol Cybern. 2012;106: 757–765. doi:10.1007/s00422-012-0514-6

3.	Santuz A, Ekizos A, Janshen L, Baltzopoulos V, Arampatzis A. On the Methodological Implications of Extracting Muscle Synergies from Human Locomotion. Int J Neural Syst. 2017;27: 1750007. doi:10.1142/S0129065717500071

4.	Janshen L, Santuz A, Ekizos A, Arampatzis A. Modular control during incline and level walking in humans. J Exp Biol. 2017;220: 807–813. doi:10.1242/jeb.148957

5.	Santuz A, Ekizos A, Janshen L, Baltzopoulos V, Arampatzis A. The Influence of Footwear on the Modular Organization of Running. Front Physiol. 2017;8: 958. doi:10.3389/fphys.2017.00958

6.	Santuz A, Brüll L, Ekizos A, Schroll A, Eckardt N, Kibele A, et al. Neuromotor Dynamics of Human Locomotion in Challenging Settings. iScience. 2020;23: 100796. doi:10.1016/j.isci.2019.100796

7.	Zumbo BD, Hubley AM. A note on misconceptions concerning prospective and retrospective power. J R Stat Soc Ser D Stat. 1998;47: 385–388. doi:10.1111/1467-9884.00139

8.	Kruschke JK, Liddell TM. The Bayesian New Statistics: Hypothesis testing, estimation, meta-analysis, and power analysis from a Bayesian perspective. Psychon Bull Rev. 2018;25: 178–206. doi:10.3758/s13423-016-1221-4

9.	Lakens D. Sample Size Justification. PsyArXiv. 2021. doi:10.31234/osf.io/9d3yf

10.	Krzywinski M, Altman N. Visualizing samples with box plots. Nat Methods. 2014;11: 119–120. doi:10.1038/nmeth.2813

11.	Allen M, Poggiali D, Whitaker K, Marshall TR, Kievit RA. Raincloud plots: a multi-platform tool for robust data visualization. Wellcome Open Res. 2019;4: 63. doi:10.12688/wellcomeopenres.15191.1

12.	Nalborczyk L, Batailler C, Loevenbruck H, Vilain A, Bürkner P-C. An introduction to bayesian multilevel models using brms: A case study of gender effects on vowel variability in standard Indonesian. J Speech, Lang Hear Res. 2019;62: 1225–1242. doi:10.1044/2018_JSLHR-S-18-0006
